# The positive effects of parents’ education level on children’s mental health in Indonesia: a result of longitudinal survey

**DOI:** 10.1186/s12889-022-13380-w

**Published:** 2022-05-12

**Authors:** Dian Fakhrunnisak, Bhina Patria

**Affiliations:** Faculty of Psychology, Universitas Gadjah Mada, Yogyakarta, Indonesia

**Keywords:** Parents’ education level, children’s mental health, Happiness, Depressive symptoms, Formal education

## Abstract

**Background:**

Mental health problems are associated with decreasing the quality of various aspects of life. Cases of mental health problems (e.g., depressive symptoms) have increased continuously. Researchers predicted depression to be the first cause of world burden diseases in 2030. One of the determinant factors of mental health is parents’ education levels, but there have been contradictory research findings. The current research investigates the effect parental education attainment has on children’s mental health.

**Methods:**

We used public data from two waves of the Indonesia Family Life Survey (IFLS) conducted in 2007 and 2014. There were 767 participants aged 15-19 years old (*M* = 16.80, *SD* = 1.37) in 2007. All participants were re-interviewed in 2014. We examined the highest level of the parents’ formal education in 2007 and the children’s mental health in 2014 to find the longitudinal effects. We used depressive symptoms and happiness as representative variables of mental health. The structural equation model (SEM) was used to examine the hypothesis, and we prioritized predictive testing over the models’ goodness of fit. We have built 12 models of combinations of children’s and parents’ sexes and different independent variables.

**Results:**

The hypothesis testing showed the longitudinal effects that fathers’ education in 2007 has on daughters’ depressive symptoms in 2014 (*β* = −.203, *p* < 0.01), while there were longitudinal effects from mothers’ education in 2007 on their daughters’ depressive symptoms (*β* = −.163, *p* < 0.01) and sons’ depressive symptoms (*β* = .096, *p* ≤ 0.05) in 2014. Testing the happiness models showed that fathers’ education in 2007 influenced the happiness of all of participants (*β* = .167, *p* < 0.01), including both sons (*β* = .206, *p* < 0.01) and daughters (*β* = 149, *p* < 0.01). On the contrary, no significant correlation was found between mothers’ education and children’s happiness across all three categories of participants.

**Conclusions:**

The general results of this study showed that parents’ education levels were associated with their children’s mental health, but there are different associations found through the different combinations of children’s and parents’ sexes.

## Background

Mental health problems impact various aspects of life, including health conditions [1], job performance [2], productivity [3], social functions [4, 5], marital quality [6], academic performance [7], and risk of suicide [8, 9]. One mental health problem, depression, became the third leading cause of world burden diseases in 2017 [10]. Murray and Lopez [11] predicted that depression would become the second leading cause in 2020 and the primary cause by 2030 [12].

One of the protective factors of mental health problems is education, which is related to general knowledge, reasoning abilities, emotional self-regulation, and interaction ability [13]. The Indonesia Basic Health Research (RISKESDAS) of 2018 shows that a higher education level is followed by a lower level of depression [14]. Additionally, the Indonesia Happiness Index of 2017 indicates that a higher education level is followed by higher happiness [15].

Mental health problem was a risk factor of poverty and vice versa that established a cycle [16]. Low education level was one fundamental factor of the cycle [17]. The limitation of job opportunities was the problem that arose from a low education level. It could cause a mental health problem and vice versa [18]. The problem caused by low education level did affect not only an individual but also the family [19]. Levels of formal education could raise the learners’ awareness about themselves and their families’ psychological development and well-being [20].

Parents’ education level is a socio-economic factor that plays a significant role in childhood mental health [21–24]. A longitudinal study by Quesnel-Vallée and Taylor [25] showed that parents’ education level predicted depression in children as they progress into adulthood. Many published studies described parents’ education level—both of fathers and mothers—as the predictors of their children’s mental health [26–29].

The study conducted by Oreopoulos and Salvanes [30] explained how parents’ education levels contribute to children’s mental health. They suggested viewing education outside of an economic context, demonstrating that education increases parenting abilities and marital quality. Samarakoon and Parinduri [31] examined the role of the mother’s educational attainment and household decision-making in Indonesia. The study showed that education improved mothers’ abilities to manage family finances, choose the best education programs for their children, control family health, and select contraception.

Previous research findings have been inconsistent [32, 33]. Park, Fuhrer [34] reported that children’s mental health has no relationship with their fathers’ educational attainment. There is a relationship, however, with mothers’ educational attainment in adulthood. Therefore, in this current study, we examined parents’ education level with two different variables: father’s and mother’s education.

In this study, we used depressive symptoms to measure mental health. Measuring depressive symptoms has been one of the most common procedures for determining mental health, as in the study conducted by Tannenbaum, Lexchin [35]. A significant advantage of using depressive symptoms to measure mental health is that it represents characteristics of multiple mental health problems over time. Its other indicator of mental health problems was the prevalence of suicide, but it could not represent all of the characteristics of common mental health problems. Tannenbaum, Lexchin [35] assumed suicide to be a reflection of severe or extreme distress conditions.

We did not use only depressive symptoms as a single indicator to measure mental health, but also incorporated happiness as the other indicator, like Diener [36], who assumed happiness to be a representation of well-being. Based on the WHO’s definition of mental health, the term does not only describe the problem or negative aspects of the field, but also the positive aspect of well-being. For this reason, researchers who have focused their studies on happiness consider happiness to reflect the power of the nation to promote better economic progress and a flourishing society [37]. The happy people had more successful life outcomes [38] such as financial, work productivity, and performance [39, 40] than unhappy people.

The current study provided a complex analysis compared to the previous one [25]. We explored longitudinal models based on sex differences of children and parents. The models analyzed two sides of mental health, such as depressive symptoms and happiness. Therefore, this study can produce more comprehensive analysis results.

## Method

We used longitudinal data from the Indonesian Family Life Surveys (IFLS), especially IFLS4 from 2007 and IFLS5 from 2014. IFLS is a longitudinal survey conducted by the RAND Corporation from 1993 until 2014 [41]. The data represents 83% of the population of Indonesia [42] and features a high number of re-contacted respondents. 90.5% of participants in IFLS4 were successfully re-interviewed in IFLS5 [41].

The IFLS used the Indonesia National Socioeconomic Survey (SUSENAS) 1993 sampling frame [41]. The sampling scheme used stratified random sampling with provinces and rural or urban locations as strata and randomly sampled within the strata. The samples of IFLS included 13 of 27 provinces by considering cost-effectiveness, thus representing 83% of Indonesia’s population [41].

Therefore, the data is ideal for testing our hypothesis. The hypothesis was the relationship between the highest level of father’s and mother’s formal education in 2007 and children’s mental health in 2014.

### Participants

The participants followed the two waves—both 2007 and 2014—of the Indonesian Family Life Surveys (IFLS). The participants’ data from the two waves were connected by PIDLINK or the ID number—which did not change from IFLS1 until IFLS5—of each participant. The final samples were 767 participants (see Table 1) based on some selection characteristics.

**Table 1:** Demographic statistics

Variables	Children	Father	Mother
	n (%)	n (%)	n (%)
**Demographic Variables**			
Sex			
Male	403 (52.5)	767 (100.0)	
Female	364 (47.5)		767 (100.0)
Education level in 2007			
Elementary school	80 (10.4)	442 (57.6)	496 (64.7)
Junior high school	225 (29.3)	120 (15.6)	149 (19.0)
Senior high school	448 (58.4)	161 (21.0)	106 (13.8)
Bachelor’s degree	14 (1.8)	44 (5.7)	19 (2.5)
Education level in 2014			
Elementary school	76 (9.9)	442 (57.6)	499 (65.1)
Junior high school	145 (18.9)	118 (15.4)	135 (17.6)
Senior high school	382 (49.8)	158 (20.6)	110 (14.3)
Bachelor’s degree	161 (21.0)	47 (6.1)	22 (2.9)
Master’s degree	3 (0.4)	2 (0.3)	1 (0.1)
Wealth in 2007			
Perceived income ladder 1, 2	161 (21.0)	189 (24.6)	207 (27.0)
Perceived income ladder 3	452 (58.9)	426 (55.5)	416 (54.2)
Perceived income ladder 4	143 (18.6)	143 (18.6)	131 (17.1)
Perceived income ladder 5, 6	10 (13.0)	7 (0.9)	13 (1.7)
Wealth in 2014			
Perceived income ladder 1, 2	125 (16.3)	196 (25.6)	189 (24.7)
Perceived income ladder 3	395 (51.5)	391 (51.0)	325 (42.4)
Perceived income ladder 4	211 (27.5)	149 (19.0)	205 (26.7)
Perceived income ladder 5, 6	36 (11.0)	32 (3.2)	46 (6.0)
**Mental Health Variables**			
Happiness			
Children’s happiness level in 2007			
Very unhappy	2 (0.3)		
Unhappy	47 (6.1)		
Happy	658 (85.8)		
Very happy	60 (7.8)		
Children’s happiness level in 2014			
Very unhappy	7 (0.9)		
Unhappy	35 (4.6)		
Happy	604 (78.7)		
Very happy	121 (15.8)		
Depressive symptoms (*mean, standard deviation*)			
Children’s depressive symptoms 2007	(M = 10.5, SD = 3.3)		
Children’s depressive symptoms 2014	(M = 13.7, SD = 4.6)		

The criteria of the inclusion were: (1) age between 15 and 19 during IFLS 4 (2007); (2) completed the necessary data (e.g., CES-D, happiness scale, and demographic data) in both survey waves (2007 and 2014); (3) the participants’ parents’ data were available in both survey waves. We merged and organized the dataset based on the characteristics using SPSS Syntax.

### Measures

#### Depressive symptoms

Depressive symptoms were measured using the Center for Epidemiologic Studies Depression (CES-D) scale. CES-D was constructed by Radloff [43] with 20 items, and modified by Andresen, Malmgren [44] to be 10 items. CES-D is a robust screening instrument of depressive symptoms in both clinical samples and non-clinical samples [45–47]. All items are rated with four-point responses, including 1 (rarely or none), 2 (some days), 3 (occasionally), and 4 (most of the time). CES-D in IFLS consists of 10 items, but we used only eight items, eliminating two items, with the positive affects considering the unidimensionality of scale [48, 49]. The sum of the points for 8 items was the depressive symptoms score in this study. The result of reliability testing found Cronbach’s alpha at 0.759.

#### Happiness

In measuring happiness using the single-item scale, respondents were asked: “Taken all things together, how would you say things are these days? Would you say you are very happy, happy, unhappy or very unhappy?” The question has already been used in national surveys like the World Value Study [50]. The participants directly evaluated their whole lives. There were four possible answers with four-point rated: very happy (4), happy (3), unhappy (2), and very unhappy (1). The single item measurement of happiness is the primary method of directly measuring the level of happiness [51–54].

#### Parents’ education level

Parents’ education level was defined by the highest level of formal education attended by the mother and father. The questionnaire inquiries about the latest formal education attended. We converted the types of formal education, for example, elementary school, secondary school, high school, or bachelor’s degree, to years of schooling. We used years of schooling to equalize types of formal educations that are equivalent in level. In the prior study [34], parental education attainment was distinguished and determined based on the father’s and mother’s educations.

#### Statistical method

The structural equation model (SEM) was used to examine the association between parents’ education level in 2007 and children’s mental health in 2014. We prioritized the predictive testing over the models’ goodness of fit. The predictive analysis focuses on estimating the independent variable’s effect on the dependent variable [55]. The significant disadvantage of the fit model testing is that it does not explain the theoretical implication of the associations between two variables because it prioritizes the goodness of fit [56] and recommend model modification [57]. The model’s modification can change the estimation of the predictive effects between variables [57, 58].

**Fig. 1:**
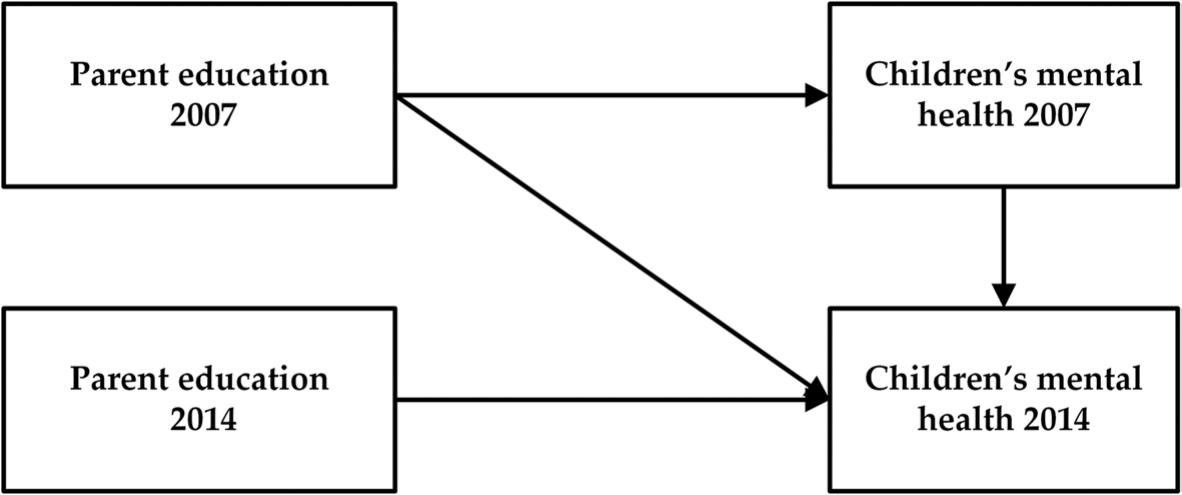
Basic model of mental health and parent’s education

**Fig. 2:**
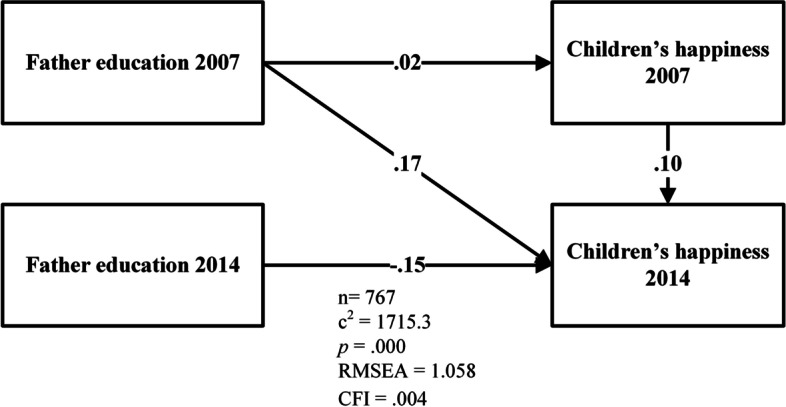
Example of the result of father’s education to children’s happiness

The hypothesis testing focused on the relationships between parents’ educations in 2007 and children’s mental health in 2014, but we kept including other variables because they contributed to individual change based on time [59]. Figure 1 was the basic model for testing each parent’s education model—mother’s and father’s education—and children’s mental health—depressive symptoms and happiness (see Fig. 2).

## Results

Table 2 presents the results of testing the 12 models. All the models were developed from the basic model (see Fig. 1). The 12 models consist of six depressive symptoms models and six happiness models that use fathers’ and mothers’ education as predictors. The models’ differentiations were based on the groups of participants and can be divided into three categories —all participants, male, and female (see Table 2).

**Table 2:** The results of hypothesis testing with structural equation model

Predictive Models	Children Data	N	***Fit Statistics of Models***			***Standardized Regression Weight***			
			χ^**2**^	RMSEA	CFI	β	SE	CR	p*
(m1) Father’s education 2007 → Children’s depressive symptoms 2014	All participants	767	1717.3	1.058	.008	.000	.053	.041	.968
(m2) Father’s education 2007 → Children’s depressive symptoms 2014	Male	403	762.3	.972	.003	.053	.074	1.079	.281
(m3) Father’s education 2007 → Children’s depressive symptoms 2014	Female	364	1021.1	1.185	.015	−.203	.076	−4.213	.000**
(m4) Mother’s education 2007 → Children’s depressive symptoms 2014	All participants	767	1829.3	1.092	.006	−.033	.065	−.931	.352
(m5) Mother’s education 2007 → Children’s depressive symptoms 2014	Male	403	958.2	1.091	.003	.096	.086	1.964	.050*
(m6) Mother’s education 2007 → Children’s depressive symptoms 2014	Female	364	869.2	1.093	.012	−.163	.097	−3.233	.001**
(m7) Father’s education 2007 → Children’s happiness 2014	All participants	767	1715.3	1.058	.004	.167	.006	4.763	.000**
(m8) Father’s education 2007 → Children’s happiness 2014	Male	403	757.3	.969	.019	.206	.008	4.282	.000**
(m9) Father’s education 2007 → Children’s happiness 2014	Female	364	1020.7	1.185	.000	.149	.008	2.957	.003**
(m10) Mother’s education 2007 → Children’s happiness 2014	All participants	767	1831.5	1.093	.005	.019	.007	.525	.600
(m11) Mother’s education 2007 → Children’s happiness 2014	Male	403	958.9	1.091	.091	.003	.009	.069	.945
(m12) Mother’s education 2007 → Children’s happiness 2014	Female	364	870.4	1.094	.000	.021	.010	.395	.693

Model 1 as m1, model 2 as m2, and so on

^*^significant < 0.05

^**^significant < 0

The criteria of goodness of fit (*n* > 250, with four observed variables) were insignificant ${\mathcal{X}}^2$, RMSEA < 0.07, and CFI ≥ 0.97 [60]. Table 2 shows that 12 models are below the recommended criteria, but we were not interested in exploring them.

Based on the testing of models concerning depressive symptoms and father’s education, we found the longitudinal effects of a father’s education in 2007 on a daughter’s depressive symptoms in 2014 (*β* = −.203, *p* < 0.01). The results of the testing of depressive symptoms and mother’s education models, showed that there were longitudinal effects of a mother’s education in 2007 on a daughter’s (*β* = −.163, *p* < 0.01) and son’s depressive symptoms (*β* = .096, *p* ≤ 0.05) in 2014. Mothers’ and fathers’ educations provided consistently negative correlations with daughters’ depressive symptoms, but mothers’ education positively correlates with depressive symptoms in their sons.

Testing the happiness models showed that fathers’ education in 2007 influenced the happiness of all participants (*β* = .167, *p* < 0.01), sons (*β* = .206, *p* < 0.01), and daughters (*β* = 149, *p* < 0.01). In the three models described, there were positive correlations between father’s education and the all participants categories. In contrast, no significant correlation was found between mother’s education and children’s happiness for all three participant categories.

## Discussion

### Father’s education and children’s happiness

This study confirmed that children’s happiness is associated with their father’s educational attainment. A possible explanation was related to fatherhood or fathering [61]. The concept of fatherhood emphasizes that the father’s participation is essential in parenting [62]. The findings from the previous study by Flouri [63] indicated that a child whose father participated in parenting had a better level of happiness than a child whose mother was the only one to participate in parenting. In brief, fatherhood associates with socio-economic and cultural contexts.

A father’s participation in their child’s health and development was more common in high-income countries than in middle- and low-income countries [64]. Fathers in low- and middle-income countries spent most of their time working to meet their families’ needs. The implication is that fathers could not know about the child’s developmental progress enough to be involved directly in parenting. In Asian cultures, traditionally, the common perception of a good father was one who could provide for the family’s financial needs [65]. The ideal understanding of a good father is the father who participates directly in the child’s care and education, taking responsibility for his child according to the child’s developmental stage [66].

The fundamental factor that encourages the father to be involved in parenting is education. Education can change the father’s view about parenting, taking it from a conventional view to a new perspective that readjusts the role of fatherhood in the family [65]. Juhari, Yaacob [67], in their study, showed that fathers with higher education had high rates of participation in any activities with their children because the high level of education motivated fathers to be open and accepting of their children. The children thought it necessary to allow their fathers to join their daily activities. The higher education fathers had better plan for supporting their children’s career, moral, spiritual, and intellectual development.

The other models’ testing results indicated no longitudinal effect of a mother’s education on children’s happiness. Those are contradictory to the results of models with a father’s education as the independent variable. The developmental perspective can explain them. When children get older, there is an increase in the determinants of happiness [68, 69]. Children need different protective factors in each developmental stage to compensate for their developmental changes.

Studies about parenting and fatherhood in Asia have showed an image of a conventional family system. Traditionally, the mother’s duty was parenting while the father’s duty was making a living—especially in a family system with patriarchal tendencies [61, 65]. When the number of fathers with higher education rose in Asia, awareness about the urgency of fatherhood also increased. The impact of active and thoughtful fatherhood increased children’s happiness [63].

### Father’s education and daughter’s level of depressive symptoms

The results—as showed in Table 2—indicated that a father’s education correlates longitudinally with his daughter’s depressive symptoms. It still related to fatherhood and the development of determinant factors of depressive symptoms in adulthood. There are different responses and outcomes between the father-daughter relationship and the father-son relationship. The father-daughter relationship could be described from a neuropsychological perspective. Studies in neuropsychology by Mascaro, Rentscher [70] explained that the father’s brain was more sensitive to recognizing the daughter’s expressions and needs than the son’s expressions. Daughters tend to be more comfortable interacting with their fathers because fathers are more critical to a son than a daughter [71].

The father-daughter relationship influences the daughter’s future emotional development, especially in her adulthood. A good father-daughter relationship is a protective factor that prevents the daughter from the risk of depression in her academics, career, a romantic relationships [71, 72]. Therefore, a father’s education is essential to fostering a good relationship because it allows the father to know his significant parenting role [61, 65].

### The opposite direction of longitudinal effects of mother’s education on children’s depressive symptoms based on children’s sexes

There are longitudinal effects of a mother’s education affecting her son’s and daughter’s depressive symptoms, but there is a positive effect on the son and a negative effect on the daughter. The results imply that the higher level of education attained by the mother is associated with a higher level of depressive symptoms for the son and a lower level of depressive symptoms for the daughter. A possible explanation for these dynamics is that higher education increases a mother’s expectations of her children [73]. Moreover, mothers have different expectations for their sons and daughters [74].

Mothers have different views and expectations about the characters of their sons and daughters [75]. The daughter is assumed to be a warm, understanding, and affectionate figure, while the son is assumed to be mature and responsible [76]. Furthermore, the son is expected to be more successful financially, able to support the family financially in the future, while the daughter becomes the person who takes care of the family [76]. The different expectations are associated with a high level of children’s depressive symptoms as they move into adulthood [77].

The general result from this study is the longitudinal effect of parents’ education level on children’s mental health, including depressive symptoms and happiness. A similar findings were found in previous works of Quesnel-Vallée and Taylor [25], Park, Fuhrer [34], and Korhonen, Remes [78]. Considering the location of the current study and previous studies, the effects of parents’ education level and children’s mental health were consistent across studies between developing and developed countries. Therefore, the parent’s education was the foundation of the family’s socioeconomic attainment, household income, and children’s mental health [25]. On the other side, the parent’s education has affected parenting skills and behavioural control so that children could grow up with a high level of well-being into adulthood [79].

This study showed the positive longitudinal effects of parents’ formal education on the children’s mental health. However, it has not identified the type of formal education of the parents. There are some models of formal education in Indonesia. The first example is formal religious education like Madrasa or formal Islamic education managed by the Ministry of Religious Affairs. We did not distinguish between religious and non-religious educations, but we focused on the level of formal education. If we associate children’s mental health with parents’ formal religious education, it might show different results. Moreover, there are equivalency programs recognized as equal to formal education.

## Conclusion

This study has found that the education level of parents is associated with their children’s mental health but has distinguished different associations when viewing the different combinations of children and parents’ sexes. We have identified three crucial findings in this study. Firstly, a father’s education has longitudinal effects on his children’s happiness, but a mother’s education has no longitudinal effects on her children’s happiness. The second finding of note is the longitudinal effect of a father’s education on his daughter’s depressive symptoms. The last finding is the different effects of a mother’s education effects on sons and daughters.

The study’s results can be used to develop targeted programs to increasing mental health by helping society understand that formal education can provide mental health benefits. We recommend increasing promotions of secondary and tertiary education and scholarship opportunities. Furthermore, Indonesia has a large area, and there are remote and border areas that do not have comprehensive educational facilities.

A key strength of the present study was using longitudinal data with 12 instances to provide specific evidence for associations. We suggest that future research examine complete associations in one model to know the more complex relations between children’s mental health and parental education attainment.

## Data Availability

The dataset analyzed in this study, IFLS Public Use Data, are available to download on the RAND website (https://www.rand.org/well-being/social-and-behavioral-policy/data/FLS/IFLS/access.html).
